# Behavioural thermoregulation via microhabitat selection of winter sleeping areas in an endangered primate: implications for habitat conservation

**DOI:** 10.1098/rsos.181113

**Published:** 2018-12-05

**Authors:** Liz A. D. Campbell, Patrick J. Tkaczynski, Mohamed Mouna, Abderrahim Derrou, Lahcen Oukannou, Bonaventura Majolo, Els van Lavieren

**Affiliations:** 1 Moroccan Primate Conservation Foundation, Azrou, Morocco; 2 WildCRU, Department of Zoology, University of Oxford, Oxford, UK; 3 Centre for Research in Evolutionary Social and Inter-Disciplinary Anthropology, University of Roehampton, London, UK; 4 Agdal Institut Scientifique, Mohamed V University, Rabat, Morocco; 5 Ifrane National Park, Azrou, Morocco; 6 School of Psychology, University of Lincoln, Lincoln, UK; 7 Conservation International Suriname, Paramaribo, Suriname

**Keywords:** Barbary macaque, conservation behaviour, Bayesian modelling, logging, thermal ecology, Atlas cedar

## Abstract

Strategic microhabitat selection allows animals in seasonally cold environments to reduce homeostatic energy costs, particularly overnight when thermoregulatory demands are greatest. Suitable sleeping areas may therefore represent important resources for winter survival. Knowledge of microhabitat use and potential impacts of anthropogenic habitat modification can aid species conservation through development of targeted habitat management plans. Wild, endangered Barbary macaques (*Macaca sylvanus*) in logged cedar-oak forest were studied to investigate (1) the hypothesis that macaques select winter sleeping areas with microhabitat characteristics that may reduce thermoregulatory costs, and, if so, (2) how to minimize damage to sleeping areas from logging. Macaques slept only in Atlas cedars (*Cedrus atlantica*). Consistent with predictions, macaques preferred sleeping in sheltered topography and dense vegetation, which may reduce exposure to wind, precipitation and cold, and preferred large trees that facilitate social huddling. This suggests that Barbary macaques employ strategic nocturnal microhabitat selection to reduce thermoregulatory costs and thus suitable sleeping areas may influence winter survival. To minimize negative impacts of logging on macaque sleeping areas, results suggest avoiding logging in topographical depressions and maintaining cedar densities greater than 250 ha^−1^ with average breast height greater than 60 cm. This study demonstrates how animal behaviour can be used to guide species-specific habitat management plans.

## 1. Introduction

Identification of resources important for a species' survival can allow targeted habitat conservation and management strategies. For animals inhabiting seasonally cold environments, energy can be a key limiting factor for winter survival [1–4]. Energy expended on thermoregulation is substantially increased by winter weather conditions, including low temperatures, strong winds and precipitation [5–7], while energy intake is often reduced due to lowered food availability by snow cover and seasonal variations in primary productivity [2,3,8,9]. Small-scale variations in habitat can produce dramatically different microclimates [5] and so reducing homeostatic energy costs through strategic microhabitat selection can increase the probability of winter survival [5,8,10]. Thermoregulatory demand is often greatest overnight when ambient temperatures reach a minimum [11] and, therefore, nocturnal microhabitat selection of winter sleeping areas can be particularly influential to the energy balance of diurnal species.

Microclimate is influenced by topography due to wind dynamics, with hilltops generally experiencing the greatest wind speeds and topographical depressions the lowest [5,12]. Vegetation also influences microclimate, as forest density affects wind speed [5] and dense overhead vegetation can impede falling precipitation and insulate against radiative heat loss to the surrounding air [5,13,14]. Social thermoregulation, i.e. grouping with other individuals to regulate body temperature, is employed by many animals to reduce cold stress [15,16], but microhabitat characteristics can limit the number of individuals able to participate, which is key to the magnitude of energy conservation that social thermoregulation confers [15]. For example, for species that sleep in caves or tree cavities, the number of participating individuals is limited by cave/cavity volume [17,18], while for species that sleep in trees, the number of individuals able to huddle together is limited by branch strength and size [19]. Strategic selection of winter sleeping areas with sheltered topography, dense vegetation and that facilitate social thermoregulation can reduce nocturnal heat loss and thus homeostatic energy costs, thereby increasing the likelihood of surviving the period of winter energy deficit. Incorporating knowledge of microhabitat characteristics used by animals to enhance survival into habitat management plans, and understanding how these microhabitat characteristics are influenced by anthropogenic activities, can aid conservation strategies.

The Barbary macaque is an endangered primate found in mountain ranges of Morocco and Algeria [20]. Anthropogenic habitat destruction is one of the greatest threats to the survival of this species [20] and the habitat of the largest remaining population in the world, the cedar-oak forests of the Moroccan Middle Atlas Mountains [20], is subject to both legal and illegal logging [21]. This species is one of the few non-human primates that inhabits a temperate environment with snowy winters, which places them under environmental pressure during winter: snow cover limits foraging opportunities [9], cold temperatures increase metabolic and physiological stress [7,22] and extreme winter conditions can dramatically increase mortality rates [23,24]. Barbary macaques sleep in trees, when available [16,25,26], and employ social thermoregulation as one mechanism to cope with cold at night [16]. Microhabitat selection of sleeping areas may be an additional behavioural strategy employed by Barbary macaques to reduce thermoregulatory demands during winter nights. If so, suitable sleeping areas may represent an important resource for winter survival which should be taken into consideration in conservation and habitat management plans.

The aims of this study were to provide the first characterization of wild Barbary macaques' sleeping areas, determine whether microhabitat selection is consistent with the hypothesis that sleeping areas contribute to winter energy conservation and are thus valuable to survival, and if so, to provide advice on how to minimize damage to these important resources from timber extraction. Sleeping areas were investigated on two scales: the areas of forest in which Barbary macaques sleep (sleeping sites) and the trees within those sleeping sites in which they sleep (sleeping trees). It was predicted that sleeping areas would be non-randomly selected with a preference for microhabitat characteristics that likely minimize nocturnal thermoregulatory demands. Specifically, it was predicted that Barbary macaques would prefer sleeping sites with topography which offers better protection from wind (i.e. valley bottoms) and avoid topographies which are more exposed (i.e. hilltops and hillsides), prefer vegetation characteristics that minimize exposure to wind, precipitation and radiative heat loss (i.e. high tree density and dense overhead vegetation), and select sleeping trees that facilitate social thermoregulation (i.e. large trees which have larger, stronger branches, allowing larger social huddles). Characteristics of sleeping sites were compared to those of samples of forest across the macaques' home range, and characteristics of sleeping trees were compared to those of samples of trees within a sleeping site, to determine if sleeping area characteristics differed from what would be expected by chance if sleeping area selection was random. Predictions were made on the probability of areas of forest being used as sleeping areas based on microhabitat characteristics, allowing development of logging advice which could be applied throughout Barbary macaque cedar-oak habitat to minimize damage to this important resource.

## 2. Methods

### 2.1. Study site

This study was conducted in Ifrane National Park, Middle Atlas Mountains, Morocco (33° 24′ N, 05° 12′ W). The forest of the study area was composed predominantly of Atlas cedar (*Cedrus atlantica*) and evergreen holm oak (*Quercus ilex*), with rare Portugese oaks (*Q. faginea*) and Montpellier maples (*Acer monspessulanum*), at an average elevation of 1800 m.a.s.l. (range 1684 m to 1924 m). The area experiences selective and illegal logging, including illegal removal of cedar branches by shepherds for livestock fodder [21,27]. The site experiences cold snowy winters and hot dry summers [9,16,21]. Data on macaques' sleeping behaviour were collected during winter (January to April 2015). Details on weather conditions during this period are provided in electronic supplementary material, table S1. Snow cover was continuous until the end of March then intermittent until the end of the study period. The study area has experienced the local extinction of Barbary macaques' natural felid predators and thus the risk of nocturnal predation in sleeping trees is low [28].

### 2.2. Subjects

Two groups of wild Barbary macaques, both fully habituated to human observers, were studied. The home ranges of these two groups did not overlap with each other but did with other groups. Blue Group consisted of 25 individuals (seven adult females, five adult males, thirteen immatures) and Green Group consisted of 42 individuals (eight adult females, seven adult males, 27 immatures). All adults were individually identified based on physical characteristics.

### 2.3. Behavioural data collection

To determine home ranges, the study groups were followed for a collective total of 113 days (56 Blue Group, 57 Green Group), recording the GPS location of the centre of the group every hour. When macaques retired to the trees in the evening, the GPS location of the group was recorded and a unique sleeping site code assigned to each area located more than 50 m from a previously recorded sleeping site. Sleeping sites were recorded for a total of 78 nights (40 Blue Group, 38 Green Group). Observers (authors LADC and PJT with three research assistants) returned in the morning before sunrise and located and identified as many sleeping macaques as possible (see Campbell *et al*. [16]). Movements during the night occur very rarely in semi-free-ranging Barbary macaques [25,26], so morning sleeping locations are assumed to be where they remained throughout the night*.* Each tree containing sleeping macaques was temporarily marked and a GPS waypoint recorded to later relocate for collection of tree measurements. If macaques were already awake and moving when researchers arrived in the morning, no sleeping tree data were recorded. Sleeping trees were recorded for a total of 56 nights (29 Blue Group, 27 Green Group).

### 2.4. Forest data collection

**Figure 1.**
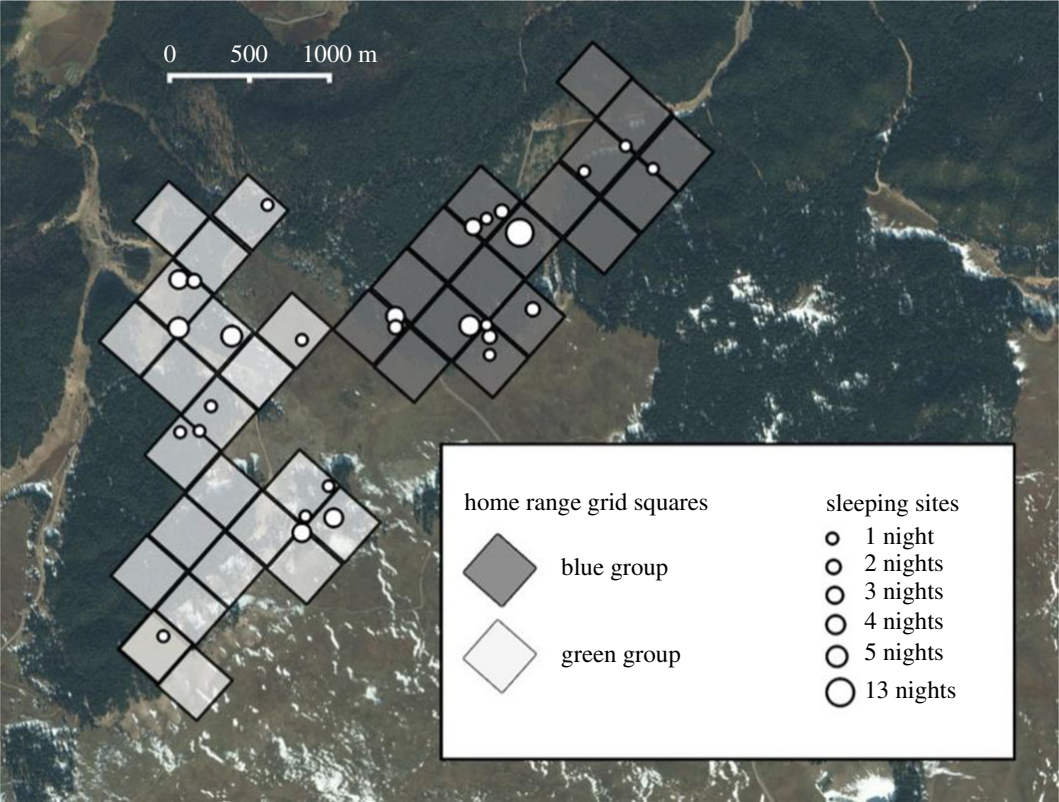
Home range grid squares (333 m × 333 m) of Blue Group (dark grey) and Green Group (light grey) of Barbary macaques in Ifrane National Park, Morocco. Locations of sampled sleeping sites are indicated with points proportional in size to the number of nights the sleeping site that was observed to have been used. A 20 m × 20 m tree plot was sampled at the centre of every home range grid square as well as at the centre of every sleeping site. Satellite base map layer by Bing Aerial © 2016 Microsoft Corporation.

The study site had been divided into a 333 m × 333 m grid for previous ecological research (unpublished, adapted from previous work [29]). To assess forest characteristics across the home range of each group, a 20 m × 20 m ‘forest tree plot' was sampled at the centre of every grid square used by the study groups, determined by the GPS ranging data (figure 1). To compare characteristics of the general forest to those of sleeping sites, a 20 m × 20 m ‘sleeping site tree plot' was sampled at the centre of every recorded sleeping site, determined by the GPS location of all recorded sleeping trees in that sleeping site. Plot size was decided based on the median area of sleeping sites (426 m^2^). The topography of each plot was recorded as flat, hilltop, hillside or valley bottom.

For every tree above 10 cm diameter at breast height (DBH) within each forest tree plot and sleeping site tree plot, as well as for all recorded sleeping trees, the tree species, DBH, and number of visible branches emerging from the trunk at the upper and lower half of the tree were recorded. The number of branches was scored as either 0 (no branches remaining, all had been cut off by shepherds or storm damaged), 1 (up to 25% of branches remaining), 2 (25–50% of branches remaining), 3 (50–75% of branches remaining) or 4 (more than 75% of branches remaining). Tree heights were also measured with the use of a clinometer, but due to collinearity with DBH, only DBH was included in the analyses. To increase reliability, author LADC collected all tree measurements, with the help of two assistants.

### 2.5. Statistical analysis

Statistical models were implemented following a Bayesian approach by Markov Chain Monte Carlo (MCMC) estimation using the JAGS software [30] through R v. 3.1.2 [31] with the packages runjags [32] and rjags [33]. All covariates were standardized to improve MCMC chain mixing [34]. A full model approach was followed [34]. Data exploration followed protocols of Zuur *et al*. [34–36] and pairwise correlations and variance inflation factors (VIF) indicated no issues of collinearity between covariates (*r*^2^ < 0.7, VIF < 2.9). All models used diffuse priors and were implemented using 3 MCMC chains with a burn-in of 10 000, a thinning rate of 10, and for enough iterations to result in a minimum effective sample size (ESS) of 10 000 samples from the posterior distribution [37]. Model scripts were adapted from Zuur *et al*. [34,35] and Kruschke [37]. MCMC chain convergence was confirmed using Gelman-Rubin statistics (all parameters between 0.99996 and 1.00009) and trace plots (electronic supplementary material, figures S1 and S2) and model validation was conducted with posterior predictive checks [34,35] (electronic supplementary material, figure S3). The posterior distribution of each parameter is provided in electronic supplementary material, figures S4 and S5, and summarized using the mean and the upper and lower bounds of the 95% highest density interval (HDI), which represents the 95% most credible values of the parameter. A parameter was considered credibly different from zero if zero was not within the 95% HDI. Graphing was performed using the R package ggplot2 [38] with code adapted from Zuur *et al*. [35].

#### 2.5.1. Topography preference and avoidance

We assessed whether the various types of topography were used for sleeping sites more or less than would be expected by chance based on their availability in the macaques' home ranges. Each forest tree plot and sleeping site tree plot had been recorded as either flat, hillside, hilltop or valley bottom. The frequency of observed use of each topography was determined by the topography of each sleeping site multiplied by the number of times each sleeping site was used. The topography of the forest tree plots was used to estimate the availability of each topography in the macaques' home ranges. If Barbary macaques are not selective in the topography used for sleeping sites, then the observed proportion of use should not differ from the proportion sampled across their home range (the expected proportion). A Bernoulli model with a vague uniform beta (1, 1) prior assessed whether there was a credible difference between the observed and expected proportions for each topography [37]. Models estimated, for each topography, the proportion of observed use, the proportion of expected use, and the difference between observed and expected use calculated at each iteration of the MCMC chains.

#### 2.5.2. Sleeping site forest characteristics

To determine whether any forest characteristics could predict whether an area of forest was used as a sleeping site, a logistic regression model with a Bernoulli distribution and logit link function was used, with sleeping site (1 = sleeping site plot, 0 = forest tree plot) as the response variable. Explanatory variables were the total number of trees, the number of Atlas cedars (the only tree species in which the subjects were observed to sleep) and the average value of each tree measurement (average DBH, average upper branching, average lower branching) for cedars in each plot.

#### 2.5.3. Sleeping tree characteristics

To assess whether trees chosen to sleep in differed from the general availability of trees at a sleeping site, a logistic mixed-effects model with a Bernoulli distribution and a logit link was used, with sleeping tree (1 = sleeping tree, 0 = non-sleeping tree) as the response variable and tree measurements (DBH, upper branching, lower branching) as the explanatory variables, with Sleeping Site ID included as a random effect. Sleeping trees that were used multiple times were included only once in the model.

Identical models as described above replacing Group ID as the response variable confirmed there was no credible difference between groups in expected or observed use of topography or tree measurements of sleeping site plots, forest tree plots or sleeping trees (see electronic supplementary material), so both study groups were pooled for all analyses.

## 3. Results

### 3.1. Home range and sleeping site sampling

The study groups collectively used 39 home range grid squares and so 39 forest tree plots were sampled (17 Blue Group, 22 Green Group). Thirty-four unique sleeping sites (15 Blue Group, 19 Green Group) were recorded over the 78 nights. For six sleeping sites, no trees were recorded because macaques were already awake when observers arrived or because of not studying the groups the following morning, so the topography of these sleeping sites was recorded, but tree plots could not be sampled, resulting in 28 sampled sleeping site plots (14 per group). A map of home range grid squares and sampled sleeping sites is shown in figure 1.

### 3.2. Reuse of sleeping sites and sleeping trees

Over the course of the study, Blue Group was observed to use 53% of identified sleeping sites (8/15) more than once and Green Group was observed to re-use 42% of identified sleeping sites (8/19). Three non-study groups were observed to use the same sleeping sites as the study groups on different nights, including two sleeping sites that were used by three different macaque groups on separate nights, and a long intergroup contest was observed between Blue Group and a non-study group in the evening at a sleeping site both groups were known to use, with the sleeping site believed to be the contested resource.

On average, sleeping locations of 44% of the group were located before the macaques awoke (average 42% Blue Group, 45% Green Group), found in an average of 5.0 ± 2.4 trees per night (4.6 ± 2.1 Blue Group, 5.4 ± 2.6 Green Group). Only Atlas cedars were used as sleeping trees. Sleeping trees were also re-used on different nights, resulting in 238 unique sleeping trees measured (116 Blue Group, 122 Green Group) from a total of 281 recorded sleeping trees (130 Blue Group, 151 Green Group). At re-used sleeping sites, an average of 34% of sleeping tree observations were in trees that were used multiple times (25% Blue Group, 41% Green Group). Displacement of a subordinate individual from their sleeping location by a dominant individual was observed on several occasions. Non-study groups were also observed to use some of the same sleeping trees as study groups on different nights.

### 3.3. Sleeping site topography preference and avoidance

Estimates of the observed proportion of use, expected proportion of use and difference between observed and expected for each topography as sleeping sites are shown in table 1. No sleeping sites or forest tree plots were recorded as ‘hilltop', so this category was omitted.

**Table 1.** Estimated parameters for each topography type for observed proportions of use as sleeping sites (*n* = 78), expected proportions of use from forest tree plot samples (*n* = 39), and the estimated difference between observed and expected use.

topography	observed mean [95% HDI]	expected mean [95% HDI]	difference mean [95% HDI]	credible non-zero difference
flat	0.475 [0.368, 0.586]	0.586 [0.437, 0.734]	−0.110 [−0.294, 0.074]	
valley bottom	0.431 [0.320, 0.546]	0.049 [0.001, 0.113]	0.382 [0.250, 0.512]	^a^
hillside	0.113 [0.050, 0.184]	0.391 [0.250, 0.541]	−0.278 [−0.441, −0.119]	^a^

^a^Credible non-zero difference between observed and expected use (0 not contained in the 95% HDI). ESS for all parameters greater than 50 000.

The proportion of time areas of flat topography that were used by macaques as sleeping sites was 0.48 (95% HDI = [0.37, 0.59]), while the expected proportion of use based on availability through sampling of forest tree plots was 0.59 (95% HDI = [0.44, 0.73]), which was not a credible difference (95% HDI = [−0.29, 0.07]). Therefore, the use of flat topography as sleeping sites did not differ from what would be expected by chance.

The estimated proportion of time valley bottoms that were used as sleeping sites was 0.43 (95% HDI = [0.32, 0.55]), whereas the expected proportion based on availability was 0.05 (95% HDI = [0.00, 0.11]), with a credible difference of 0.38 (95% HDI = [0.25, 0.51]). Valley bottoms were therefore used more than would be expected by chance based on their availability in the macaques' home ranges.

The estimated proportion of use of hillsides as sleeping sites was 0.11 (95% HDI = [0.05, 0.18]), whereas the expected proportion of use based on availability was 0.39 (95% HDI = [0.25, 0.54]), with a credible difference of −0.28 (95% HDI = [−0.44, −0.12]). Hillsides were used as sleeping sites less than would be expected by chance.

### 3.4. Sleeping site forest characteristics

**Figure 2.**
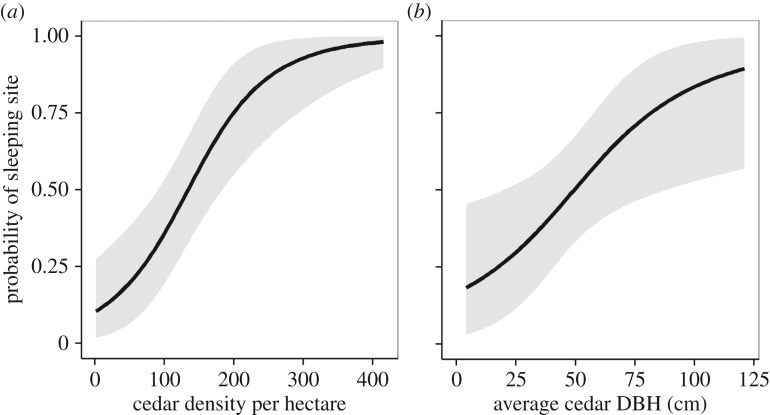
Logistic curves showing the predicted probability (posterior distribution mean ± 95% HDI) of an area of forest being used as a sleeping site by Barbary macaques as a function of (*a*) Atlas cedar density (ha^−1^) and (*b*) average Atlas cedar diameter at breast height (DBH, cm), given average values of the other covariates.

Estimated regression parameters from the logistic model, assessing whether any forest variables could credibly predict whether an area was a sleeping site or general forest, are presented in table 2. Ten forest tree plots that contained no trees because of being at the forest edge were excluded from the model because of being unsuitable as potential sleeping sites. Variables with slopes credibly different from zero were density of cedars (95% HDI = [0.77, 2.92]) and average DBH of cedars (95% HDI = [0.15, 2.02]); as the value of these variables increased, an area was more likely to be a sleeping site. Figure 2 shows the fitted values for these two variables using the mean of the posterior distribution at average values of the other covariates. This shows that areas of forest with a cedar density lower than 200–250 trees ha^−1^ and average cedar size below approximately 60 cm DBH are unlikely to be used as sleeping sites. There was no credible difference between sleeping site plots and forest tree plots in total tree density or the average amount of branching in the top or bottom half of trees. The same conclusions were reached when only a subset of the data with ‘flat' topography was analysed, removing potential confounding effects of topography with forest characteristics (see electronic supplementary material, table S4 and figure S5).

**Table 2.** Standardized regression parameter estimates from a logistic model comparing variables from sleeping site tree plots (*n* = 28) to forest tree plots (*n* = 29), estimated following a Bayesian approach, including the mean, standard deviation (s.d.) and 95% highest density interval (HDI) of the posterior distribution.

variable	mean	s.d.	2.5% HDI	97.5% HDI	ESS	credible non-zero effect
intercept	−0.133	0.370	−0.889	0.558	31 035	
cedar density	1.808	0.552	0.782	2.919	29 821	^a^
total tree density	−0.910	0.478	−1.858	0.010	20 646	
average DBH	1.043	0.476	0.146	2.002	29 586	^a^
average upper branching	−0.474	0.608	−1.689	0.691	30 000	
average lower branching	−0.520	0.554	−1.630	0.561	30 000	

^a^Credible non-zero effect (0 not contained in the 95% HDI).

### 3.5. Sleeping tree characteristics

**Figure 3.**
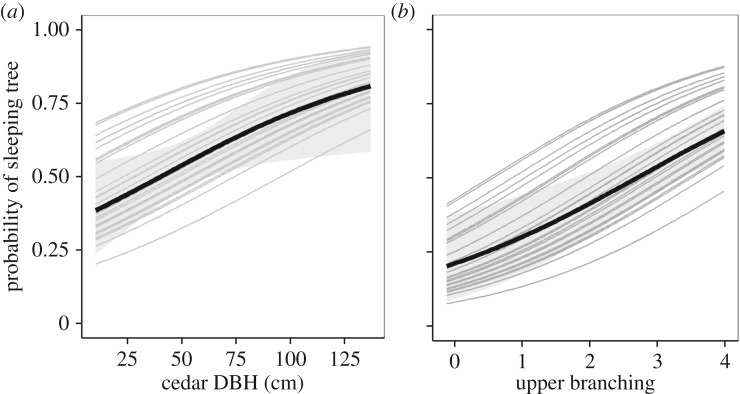
The probability that an Atlas cedar will be used by Barbary macaques as a sleeping tree as a function of (*a*) DBH (cm) and (*b*) amount of branches in the upper half of the tree (0 = 0% remaining, all branches had been cut off or storm damaged, 4 = 75–100% of branches remaining), given average values of the other covariates in the model. Random variation between sleeping sites is indicated by the grey lines with each representing a different sleeping site, and the black line shows the average of all sleeping sites. The 95% HDI around the mean of all sleeping sites is shown by the shaded grey area.

Estimated regression parameters for the logistic mixed effect model comparing sleeping trees to non-sleeping trees within a sleeping site are presented in table 3. The variables able to credibly predict whether a tree was slept in were DBH (95% HDI = [0.04, 0.59]) and branching on the upper half of the tree (95% HDI = [0.23, 0.73]); as the value of these variables increases, the probability that a tree will be used as a sleeping tree increases (figure 3). Branching on the lower half of the tree could not credibly predict if a tree was used as a sleeping tree.

**Table 3.** Standardized regression parameter estimates from a Bayesian logistic mixed effect model comparing variables from sleeping trees (*n* = 238) to the general availability of trees (*n* = 189) within a sleeping site (*n* = 28), including the mean, standard deviation (s.d.), upper and lower bounds of the 95% highest density interval (HDI) and the effective sample size (ESS) of the posterior distribution.

variable	mean	s.d.	2.5% HDI	97.5% HDI	ESS	credible non-zero effect
intercept	0.224	0.166	−0.104	0.549	30 000	
DBH	0.316	0.140	0.042	0.590	30 296	a
upper branching	0.484	0.129	0.230	0.732	30 000	a
lower branching	0.043	0.118	−0.185	0.276	31 202	
random effect variance	0.610	0.193	0.251	0.998	28 706	a

^a^Credible non-zero effect (0 not contained in the 95% HDI).

## 4. Discussion

### 4.1. Non-random use of sleeping areas

Multiple evidence was found of sleeping area preference in wild Barbary macaques: particular sleeping sites and sleeping trees were used repeatedly by the study groups, the same sleeping sites and sleeping trees were used by different macaque groups on separate nights, a fight between two groups presumably over a sleeping site was observed, and within-group displacement of subordinate individuals by dominant macaques over sleeping locations was observed. This suggests that some areas are superior to others in terms of quality and that high-quality sleeping areas are a resource worth fighting over.

### 4.2. Topography preference and avoidance

Relative to the availability of these topographies in the groups' home ranges, valley bottoms were used as sleeping sites more than would be expected, hillsides were used less than would be expected, and flat areas were used in proportion to their availability. Topography can substantially influence wind dynamics and thus mammal energetics, as exposure to high winds at low temperatures results in substantial heat loss and thus energy cost required to maintain body temperature [5,10]. For example, at a constant air temperature, wind exposure increases energetic costs of small mammals by 10–20% [10], and in red deer (*Cervus elaphus*), limiting wind exposure through use of sheltered topography can reduce heat loss by half [5]. Sheltered valleys can considerably reduce wind flow, whereas hillsides experience greater wind flow [5,12]. The preference for valley bottoms and avoidance of hillsides as winter sleeping sites is likely an adaptive strategy by Barbary macaques to reduce nocturnal energy costs through a reduction in wind exposure. Similar results have been reported in other temperate primates [19,39–43].

### 4.3. Tree vegetation preferences

It was hypothesized that Barbary macaques would exploit vegetation characteristics which promote warmer microclimates through protection from wind, precipitation and radiative heat loss. Macaques only slept in Atlas cedars, sleeping sites had higher cedar densities than samples of the general forest in the macaques' home ranges, and sleeping trees had more branches in the upper half of the tree compared to non-sleeping trees at a sleeping site.

The preference for sleeping sites with high cedar densities may reduce convective heat loss, as tree density influences wind exposure [5]. Only cedar density, not total tree density (cedars and oaks), influenced sleeping site selection. The cedars at the study site are much taller than the oaks (average cedar height = 11.9 m, *N* = 368, average oak height = 6.1 m, *N* = 424; LAD Campbell *et al*., 2015 unpublished data), so increased oak density would do little to block wind at the height of sleeping macaques. Furthermore, because subjects slept only in cedars, they likely require a high density of suitable trees to ensure group cohesion is maintained at night [41]. The preference for sleeping trees with many upper branches may provide protection from falling rain and snow and reduce radiative heat loss. Exposure to precipitation at low temperatures can be very detrimental to heat conservation due to evaporative cooling, reduction of fur insulation and amplified effects of windchill [6]. Rain increases Barbary macaque hormone levels indicative of metabolic rate [7] and Barbary macaques increase the size of social thermoregulation huddles in response to precipitation and low temperatures [16], suggesting precipitation imposes substantial thermoregulatory costs. Reducing exposure to winter precipitation through selection of sleeping trees with many upper branches therefore likely confers energetic benefits. Dense canopies also decrease radiative heat loss to the cold night sky [5,13]. Other temperate primates also sleep under dense canopies in winter [19], apparently for the thermoregulatory benefits*.* Only the number of branches in the upper half of the tree and not the lower half differed between sleeping trees and other trees at a sleeping site, possibly because branches below sleeping monkeys would not protect from precipitation or radiative heat loss.

Barbary macaques slept only in Atlas cedars and never in the available evergreen oaks. Other temperate primates also show a preference for sleeping in conifers [19,42–44]. Coniferous forests maintain higher nocturnal air temperatures than other forest types [14] and conifers are particularly good at impeding falling snow due to the accumulation of snow on upper branches. Various species of deer increasingly use areas of dense conifer forest as snow depth increases due to the precipitation-sheltering effect [13,45] and Japanese macaques sleep only in conifers when it snows*,* though they will sleep in deciduous tree species when it does not [19].

### 4.4. Facilitation of social thermoregulation

Huddling with conspecifics can substantially reduce heat loss by reducing body surface area exposed to the cold [15], but physical constraints can limit the number of individuals able to participate [46], a key factor in the magnitude of energetic benefit [15]. A concurrent study found that Barbary macaques huddle with conspecifics as a form of social thermoregulation, forming larger huddles in response to greater thermoregulatory challenges [16]. Sleeping on tree branches, Barbary macaque huddle sizes are likely restricted by branch size and strength [16,19,46]. This is supported by findings that although Barbary macaques increase huddle sizes in response to poor weather, huddles are small and rarely exceed three adults [16], yet much larger huddles can form in other macaques when sleeping on the ground [47,48]. Results supported the prediction that larger trees, and thus larger branches, would be selected to maximize the size of social thermoregulation huddles, as Barbary macaques selected sleeping sites with larger average tree diameter than the general forest and selected sleeping trees with larger diameter than other trees in the sleeping site.

### 4.5. Factors aside from thermoregulation which may be involved in sleeping site selection

Although results support the hypothesis that winter sleeping area selection contributes to thermoregulation, other factors may also play a role [46,49]. Despite current predation risk in sleeping trees being low [28], some results could be interpreted as consistent with predator avoidance. Dense vegetation improves concealment and large trees may be more difficult for predators to access [46,49]. Using multiple sleeping sites but reusing some several times may reflect a compromise between being unpredictable to predators but being familiar with a location and thus improving chances of escape [46,49]. However, if reducing detection was a primary concern, monkeys may be expected to also select for many lower branches to improve concealment from the ground, rather than only upper branches.

Parasite avoidance, distance to food sources and interactions with conspecific groups may also influence Barbary macaque sleeping area selection. If only thermoregulation was important, macaques would sleep only at the ‘best' site. Instead, using many sites reduces the risk of parasitic infection [50] and can maximize access to food sources across a group's home range while minimizing travel costs [39,51]. This may be supported by the finding that the group with the larger home range also used more sleeping sites, which would limit travel costs between feeding areas and sleeping sites. Proximity of sleeping sites to food sources may be particularly important to Barbary macaques during winter as they experience reduced food availability [9,52] and time feeding increases winter survival [23]. Because different groups used the same sleeping sites and an extended intergroup contest over a sleeping site was observed, intergroup competition may also influence Barbary macaque sleeping area selection [42,53,54].

Sleeping area selection is likely a trade-off between several competing factors [43,46,49]. Although thermoregulation is thought to be the greatest factor in Barbary macaque winter sleeping area selection because winter mortality is a greater risk to survival than predation or parasitic infections [23,24], other factors may also be involved and warrant further investigation.

### 4.6. Implications for conservation and habitat management

The loss of habitat due to human activities is a major issue facing wildlife conservation globally. To develop focused habitat management plans, it is important to take into consideration the specific requirements of the species. The hypothesis that winter sleeping areas are selected to minimize nocturnal energetic costs was supported, suggesting suitable sleeping areas may be important resources for Barbary macaque winter survival. Sleeping areas of suitable quality and quantity may also contribute to survival through predator avoidance, reduced parasite exposure and minimizing distance to food sources.

Both selective and illegal logging occur in Barbary macaque habitat [21,27] and characteristics of winter sleeping areas preferred by Barbary macaques, possibly for their thermal benefit, are influenced by logging. The illegal removal of cedar branches by shepherds for livestock fodder [21,27] is detrimental to macaques' preference for cedars with many upper branches and may reduce trees' capacity to shelter macaques from precipitation, wind and radiative heat loss. The preference for sleeping sites with high densities of large cedars is impacted by the removal of trees by logging which reduces tree density and can reduce stand size if large- and medium-sized trees are targeted for extraction [21]. Knowledge of Barbary macaque sleeping area selection, as presented in this study, can be used to guide management plans to minimize the impact of human resource exploitation on Barbary macaque habitat. Placement of logging concessions on hillsides, and perhaps hilltops, may be least likely to damage Barbary macaque winter sleeping sites, while topographical depressions should be avoided. Areas logged below a density of approximately 200–250 cedars ha^−1^ and with an average cedar DBH below approximately 60 cm are unlikely to be used as sleeping sites, so maintaining selective logging above these minimums may minimize damage to macaque sleeping areas in Ifrane National Park. Previous research in Ifrane National Park [21] found that cedar density and average DBH in undisturbed forest and previously logged forest approximates these estimates, but that highly disturbed forest appears to be unsuitable for Barbary macaque sleeping areas due to a lack of large enough cedars (average cedar DBH 36.6 cm [21]). This study was conducted only during winter and so seasonal variations in sleeping area selection was not assessed. Harsh winters can result in considerable mortality in Barbary macaques [23,24] and thus it is most valuable for the conservation of this endangered species that resources that help macaques survive winter are identified and protected. Similar research could be conducted in other locations where timber extraction conflicts with primate conservation to determine how primates exploit microhabitat characteristics to enhance survival and estimate minimum requirements so that suitable areas are preserved.

## Supplementary Material

Supplementary Material

## Data accessibility

Data are available from the Dryad Digital Repository at: http://dx.doi.org/10.5061/dryad.sb54c59 [55].
