# Correction to: ‘Behavioural thermoregulation via microhabitat selection of winter sleeping areas in an endangered primate: implications for habitat conservation’

**DOI:** 10.1098/rsos.190045

**Published:** 2019-01-30

**Authors:** Liz A. D. Campbell, Patrick J. Tkaczynski, Mohamed Mouna, Abderrahim Derrou, Lahcen Oukannou, Bonaventura Majolo, Els van Lavieren

*R. Soc. open sci.* **5**, 181113. (Published 27 November 2018). (doi:10.1098/rsos.181113)

This correction refers to an error in the abstract. The acronym DBH, meaning ‘diameter at breast height’, was accidentally changed to ‘breast height’, therefore the key word *diameter* was missing. The correct text appears below.

To minimize negative impacts of logging on macaque sleeping areas, results suggest avoiding logging in topographical depressions and maintaining cedar densities greater than 250 ha^−1^ with average diameter at breast height greater than 60 cm.

