# Supplementary material for: Behavioural thermoregulation via microhabitat selection of winter sleeping areas in an endangered primate: implications for habitat conservation
Source: R Soc Open Sci. 2018 Dec 5;5(12):181113. doi: 10.1098/rsos.181113 (PMC6304142; doi:10.1098/rsos.181113)
Supplement: Supplementary Material [file rsos181113supp1.pdf]

Supplementary Materials

Behavioural thermoregulation via microhabitat selection of winter sleeping areas in an endangered primate: Implications for habitat conservation

Liz A. D. Campbell[1,2], Patrick J. Tkaczynski[3], Mohamed Mouna[4], Abderrahim Derrou[5], Lahcen Oukannou[5], Bonaventura Majolo[6], Els van Lavieren[1,7]

[1]Moroccan Primate Conservation Foundation, Azrou, Morocco
[2]WildCRU, Department of Zoology, University of Oxford, Oxford, United Kingdom
[3]Centre for Research in Evolutionary Social and Inter-Disciplinary Anthropology, University of Roehampton, London, United Kingdom
[4]Agdal Institut Scientifique, Mohamed V University, Rabat, Morocco
[5]Ifrane National Park, Azrou, Morocco
[6]School of Psychology, University of Lincoln, Lincoln, United Kingdom
[7]Conservation International Suriname, Paramaribo, Suriname

Corresponding author Liz A. D. Campbell, lizadcampbell@gmail.com

Table S1: Weather measurements (± SD, where applicable) over the study period. Values obtained for Azrou, Morocco (1250 m a.s.l.) from www.meteoblue.com for 1st January to 16th April, 2015.

Month	Average overnight temperature (°C)	Minimum overnight temperature (°C)	Average overnight precipitation (mm)	Average overnight snowfall (mm)	Average overnight wind speed (m/s)	Maximum overnight wind speed (m/s)
**January**	6.33 ± 3.47	-2.6	3.80 ± 7.05	1.16 ± 2.83	4.57 ± 2.74	14.2
**February**	4.24 ± 2.28	-3.7	1.02 ± 2.11	0.34 ± 0.92	4.03 ± 2.89	18.2
**March**	9.30 ± 3.73	-0.2	2.17 ± 5.02	0.51 ± 1.69	3.45 ±1.68	10.9
**April**	13.67 ± 2.83	7.0	0.94 ± 3.22	0.00 ± 0.00	2.62 ± 1.11	6.2

Table S2: Results from logistic models assessing whether there was a credible difference between Blue Group (BG) and Green Group (GG) in either the observed or expected proportion of use of areas with different topographies as sleeping sites, estimated following a Bayesian approach.

Topography	BG Mean [95% HDI]	GG Mean [95% HDI]	Difference Mean [95% HDI]	Credible Non-Zero Difference
Observed Use				
**Flat**	0.523 [0.376, 0.671]	0.425 [0.274, 0.574]	0.098 [-0.117, 0.307]	No
**Valley**	0.429 [0.282, 0.577]	0.425 [0.274, 0.577]	0.003 [-0.214, 0.208]	No
**Hillside**	0.072 [0.009, 0.149]	0.175 [0.066, 0.292]	-0.103 [-0.248, 0.034]	No
Expected Use				
**Flat**	0.632 [0.420, 0.834]	0.541 [0.345, 0.732]	0.090 [-0.205, 0.371]	No
**Valley**	0.105 [0.003, 0.238]	0.042 [0.000, 0.122]	0.063 [-0.080, 0.240]	No
**Hillside**	0.315 [0.123, 0.518]	0.458 [0.264, 0.651]	-0.143 [-0.415, 0.145]	No

Table S3: Standardized parameter estimates from logistic regression models assessing whether there was a credible difference between the two study groups in any variables relating to sleeping site plots, forest tree plots, or sleeping trees, estimated following a Bayesian approach.

Variable	Mean	SD	2.5% HDI	97.5% HDI	ESS	Credible Non-Zero Difference
Sleeping Site Plots						
**Intercept**	-1.116	0.697	-2.651	0.165	27310	No
**Cedar Density**	-0.872	0.842	-2.552	0.761	27555	No
**Total Tree Density**	1.218	0.726	-0.136	2.681	26289	No
**Average DBH**	-0.572	0.650	-1.888	0.698	27587	No
**Average Upper Branching**	-0.307	1.239	-2.742	2.170	27199	No
**Average Lower Branching**	2.416	1.334	-0.095	5.079	27387	No
Forest Tree Plots						
**Intercept**	-0.070	0.557	-1.151	1.053	26977	No
**Cedar Density**	-0.802	0.768	-2.355	0.668	28127	No
**Total Tree Density**	1.353	0.803	-0.166	2.946	26835	No
**Average DBH**	-0.686	0.764	-2.196	0.832	27136	No
**Average Upper Branching**	-0.375	1.529	-3.334	2.665	26453	No
**Average Lower Branching**	2.865	1.589	-0.226	5.981	26114	No
Sleeping Trees						
**Intercept**	-0.559	2.168	-4.832	3.701	25058	No
**DBH**	-0.131	0.885	-1.898	1.581	29800	No
**Upper Branching**	0.175	0.908	-1.583	1.970	30000	No
**Lower Branching**	-0.156	0.880	-1.882	1.568	30000	No
**Random Effect Variance**	9.220	0.689	7.819	10.000	29256	*

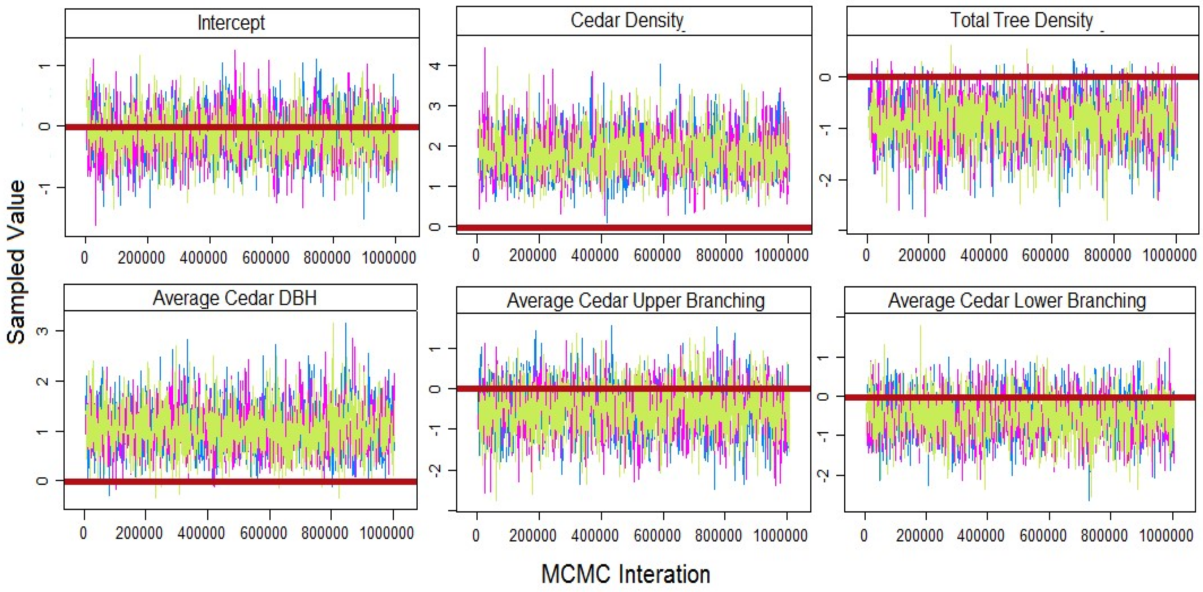

Figure S1: MCMC chain traceplots from logistic GLM assessing the difference in forest characteristics between sleeping sites and forest plots. A sampled parameter value of zero is indicated by the red line. ESS for all parameters > 29,000.

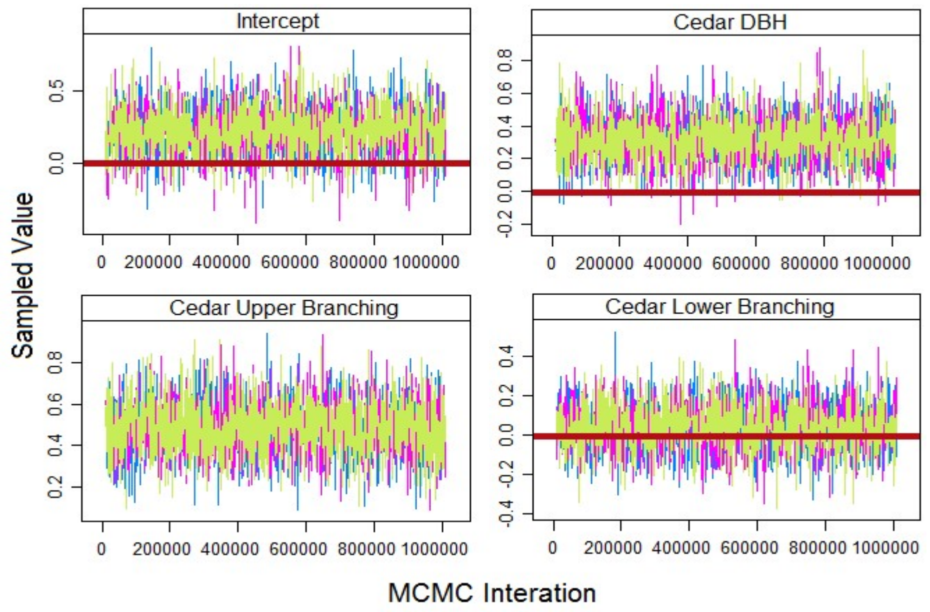

Figure S2: MCMC chain traceplots from logistic GLMM assessing differences between sleeping trees and non-sleeping trees within a sleeping site. A sampled vale of zero is indicated by the red line. ESS for all parameters > 30,000.

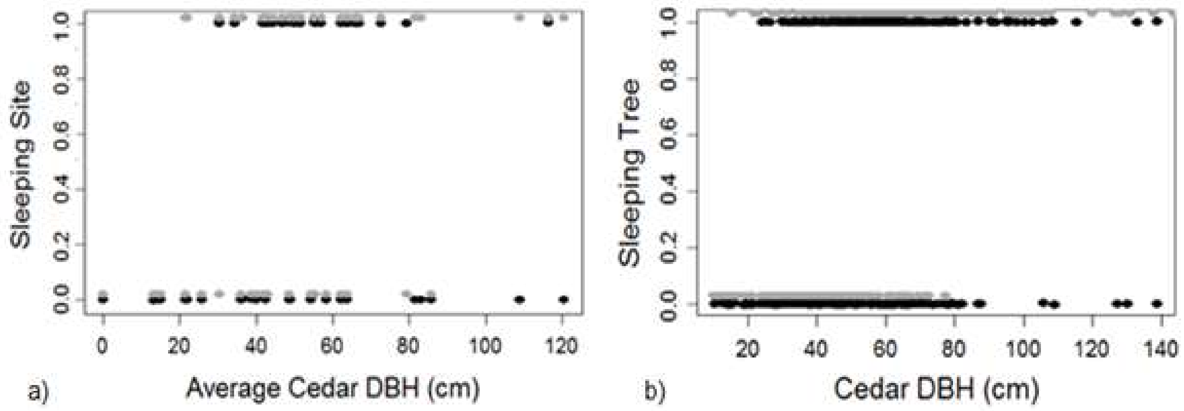

Figure S3: Posterior predictive check of (a) Logistic GLM comparing sleeping site tree plots to forest tree plots and (b) Logistic GLMM comparing sleeping trees and non-sleeping tree within a sleeping site, including the raw data points (black) and simulated data points from the fitted model (grey). The simulated values are shown slightly above the raw data and random jittering has been added along the x-axis to aid visualization.

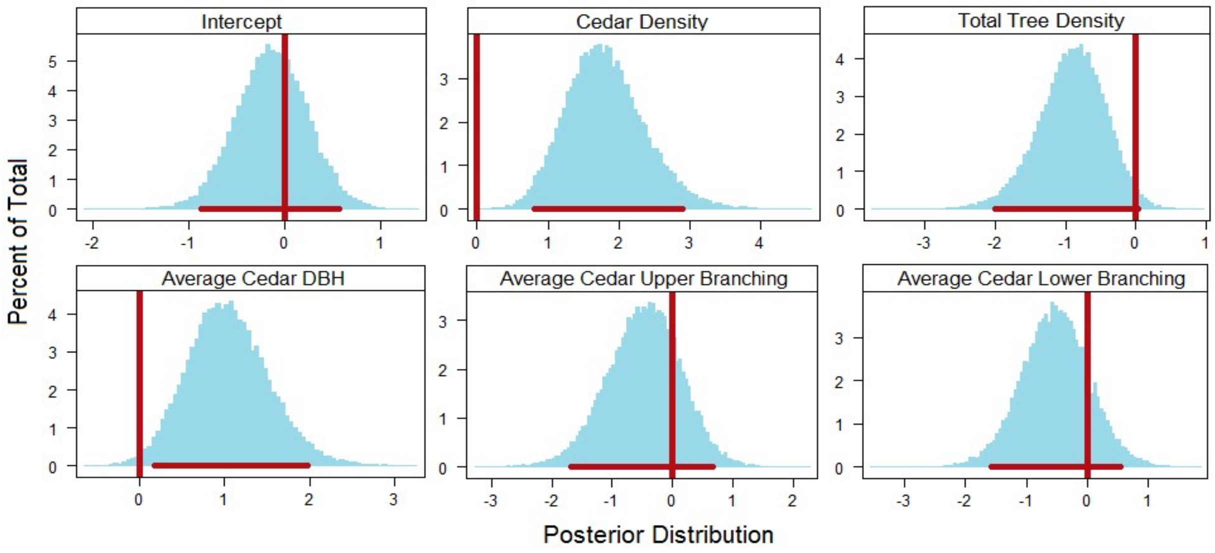

Figure S4: Posterior distributions from logistic GLM assessing the difference in forest characteristics between sleeping sites and forest plots, including the 95% highest density interval. A parameter value of zero is indicated by the vertical red line.

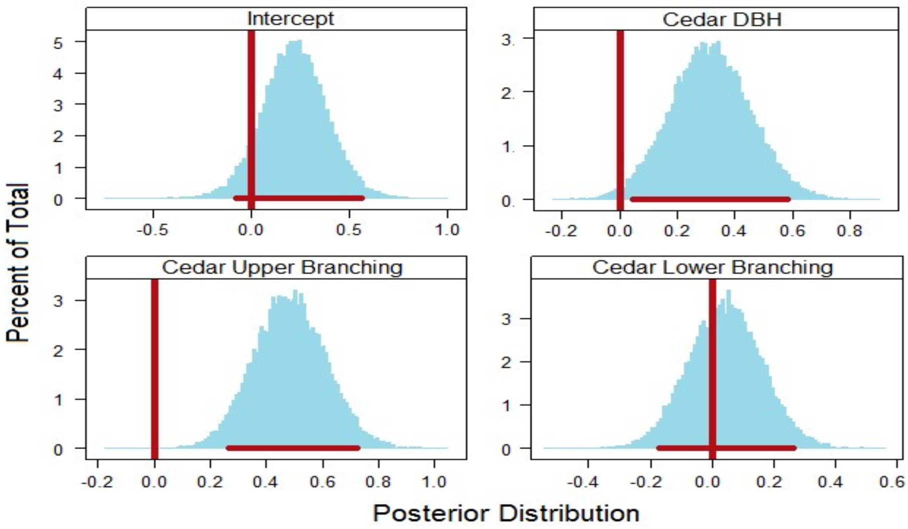

Figure S5: Posterior distributions from GLMM assessing differences between sleeping trees and non-sleeping trees within a sleeping site.

Table S4: Standardized regression parameter estimates from logistic model comparing a subset of sleeping tree plots and forest tree plots with only flat topography (n=32), estimated following a Bayesian approach, including the mean, standard deviation (SD) and 95% highest density interval (HDI) of the posterior distribution.

**Variable**	**Mean**	**SD**	**2.5% HDI**	**97.5% HDI**	**ESS**	**Credible Non-Zero Effect**
Intercept	-0.814	1.002	-2.840	1.091	26066	
Cedar Density	3.055	1.109	1.040	5.258	23187	*
Total Tree Density	-2.056	1.508	-5.135	0.457	24891	
Average DBH	1.845	0.940	0.058	3.684	25091	*
Average Upper Branching	-0.669	1.062	-2.814	1.377	27331	
Average Lower Branching	-1.347	1.059	-3.496	0.643	26016	

* Credible non-zero effect (0 not contained in the 95% HDI)

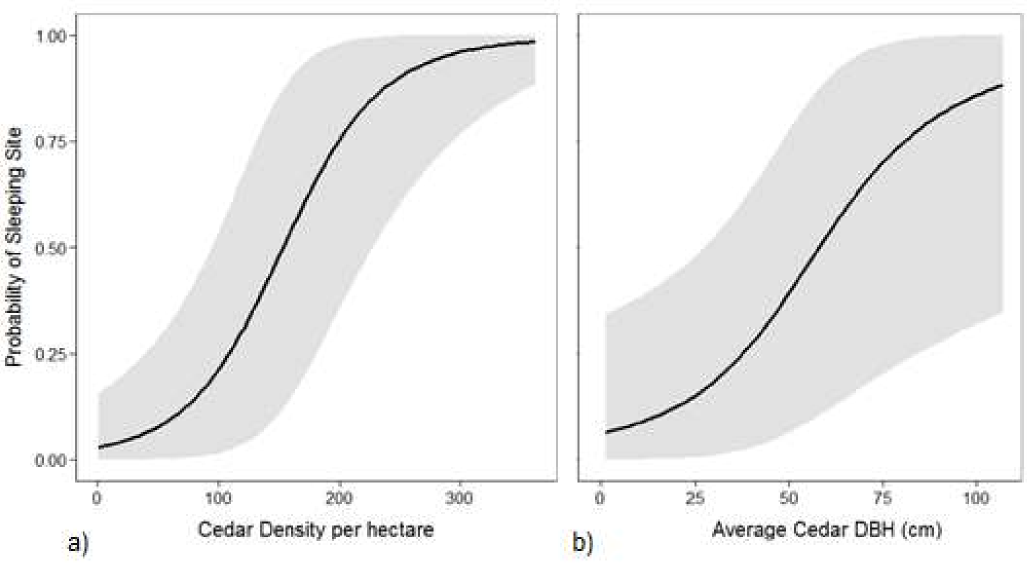

Figure S6: Logistic curves showing the predicted probability (posterior distribution mean ± 95% HDI) of an area of forest being used as a sleeping site by Barbary macaques as a function of a) Atlas cedar density (ha^{-1}) and b) average Atlas cedar diameter at breast height (DBH, cm), given average values of the other covariates, when considering only a subset of the data with flat topography.
